# Microbial Communities Vary by Body Region but Remain Consistent Across Color Morphs in the Solitary Ascidian *Rhopalaea abdominalis*

**DOI:** 10.1155/ijm/1675944

**Published:** 2026-08-29

**Authors:** Brenna Hutchings, Susanna López-Legentil, Lauren M. Stefaniak, Marie L. Nydam, Patrick M. Erwin

**Affiliations:** ^1^ Department of Biology & Marine Biology, Center for Marine Science, University of North Carolina Wilmington, Wilmington, North Carolina, USA, uncw.edu; ^2^ Department of Marine Science, Coastal Carolina University, Conway, South Carolina, USA, coastal.edu; ^3^ Life Sciences Concentration, SOKA University of America, Aliso Viejo, California, USA, soka.edu

**Keywords:** ASV, bacteria, microbiome, OTU, sea-squirt, tunicate

## Abstract

Ascidians are filter‐feeding marine invertebrates that display inter‐ and intraspecific color variation and host diverse microbiomes. Although color variation has been used as an indicator of speciation and correlated with distinct microbiome structure in some colonial species, there is limited research on microbiome variation across color morphs and body regions in solitary ascidians. To address these knowledge gaps, three color morphs (purple, orange, and intermediate shades of pink) of the solitary Belizean ascidian *Rhopalaea abdominalis* were collected for phylogenetic analysis using the Cytochrome Oxidase I (COI) gene and for microbial characterization via sequencing the V4 region of the 16S rRNA gene. Microbial sequences derived from the ascidian branchial sac, gut, and tunic body regions were processed using both operational taxonomic unit (OTU) and amplicon sequence variant (ASV) pipelines to determine the resolution and consistency of sequence processing methods using mothur and QIIME 2 software, respectively. COI sequencing revealed two clades that were independent of color morphs. Microbiome characterization showed no significant differences in alpha‐ or beta‐diversity across ascidian color morphs or COI clades but distinct microbiomes within each body region. The branchial sac harbored a higher number of core members (i.e., detected across all host individuals) that included known ascidian symbionts (e.g., *Endozoicomonas*), whereas the gut and tunic were colonized by taxa identified in seawater and across multiple classes of marine invertebrates. Results were highly congruent between OTU‐ and ASV‐based pipelines, yielding statistically equivalent alpha‐diversity metrics, consistent beta‐diversity patterns, and similar core membership profiles. These findings reinforce the validity of both methods for studying microbial symbiont communities and corroborate previous research showing distinct microbiomes by body region and overall conservation when color morphs lack genetic differentiation.

## 1. Introduction

Ascidians or sea‐squirts (phylum: Chordata; class: Ascidiacea) are filter‐feeding, benthic marine invertebrates that display an array of colorations in both natural and artificial habitats. These colorations are due to concentrations of pigments held within blood cells that accumulate in tissues like the branchial sac (filter‐feeding apparatus) and the tunic (outer cellulose layer) [1]. Vibrant shades of violet, blue, orange, and red are caused by pigments that are sometimes named for their association with ascidians, like the tunichrome and tunichlorin pigments. Pigments can also be named for a particular species, such as eudistomin and didemnimides in species of *Eudistoma* and *Didemnum*, respectively [2]. Surface coloration is a conspicuous feature commonly used to describe and differentiate ascidian species; however, pigmentation can vary among individuals within the same species.

Genetic identification of ascidians aids in discerning whether color morphs represent multiple, distinct species (i.e., cryptic speciation) or phenotypic variants of the same species (i.e., phenotypic plasticity). For example, the orange and yellow‐gray color morphs of the colonial *Pseudodistoma crucigaster* are likely two separate species that arose following an ancient habitat fragmentation event [3]. Similarly, white and green‐yellow color morphs of *Trididemnum orbiculatum* are proposed to be separate species that became reproductively isolated due to predation patterns and asynchronous spawning [4]. Orange and beige colonies are also likely different species previously considered *Didemnum granulatum* [5]. In other studies, color variation within ascidian species did not correspond to distinct genetic lineages, including *Cystodytes dellechiajei* [6], *Botrylloides diegensis* (misidentified in the field as *B. violaceus* based on color morphology) [7], and *Polyclinum constellatum* [8].

Ascidian species host diverse microbiomes (bacteria, archaea, and fungi) [9, 10], and pigment concentration in ascidian tissues could impact, or be impacted by, the presence of bacterial symbionts. For example, the high concentrations of pigments in dark‐gray colonies of *Didemnum molle* are hypothesized to protect against ultraviolet radiation, leading to higher abundances of the mutualistic, photosynthetic bacterium *Prochloron* [11]. In the colonial species *Trididemnum solidum*, however, colony color is due to the photosynthetic pigments produced by symbionts, with dark purple colonies having significantly higher concentrations of phycoerythrin [12]. To date, symbiont contributions to ascidian coloration are restricted to photosynthetic taxa, whereas overall symbiont communities appear to correspond with host lineage rather than color morph alone. In *Distaplia bermudensis*, for example, colonies with light tunics and dark zooid openings and colonies with dark tunics and light zooid openings are genetically distinct lineages that also host significantly different microbial communities [13]. In *Polyclinum constellatum*, however, green and red colonies formed a single phylogenetic clade with no significant differences in microbiome diversity and composition [8]. Thus, when host coloration has a genetic basis, it can indicate speciation and unique microbial structure in colonial ascidians.

Few published studies have explored color variation in solitary ascidians. Red, pink, and white color morphs of the edible *Halocynthia roretzi* correspond to the same species, given similar morphological traits and inconclusive alloreactivity tests, as a proxy for testing genetic divergence between color morphs [14]. Conversely, *Pyura stolonifera* has been subdivided into four species based on genetic and morphological characters, including differing tunic textures and colors [15]. There is currently no research investigating the correlation between host color morph and microbiome in a solitary ascidian species. To fill this knowledge gap, three color morphs (purple, orange, and intermediate) of the aplousobranch *Rhopalaea abdominalis* were collected from Belizean reef sites to investigate the genetic and microbial correlates of color variation. The microbial communities present in the branchial sac, gut, and tunic of *R. abdominalis* color morphs were characterized using both the mothur software package to group sequences into operational taxonomic units (OTUs) based on 97% sequence similarity (3% sequence dissimilarity) [16] and the QIIME 2 software package to assign sequences to unique amplicon sequence variants (ASVs) using the DADA2 pipeline [17]. Debate continues over which of these two popular pipelines provides higher quality results [18, 19], with suggestions that a combination of clustering and denoising is preferable to selecting one method over the other in Cytochrome Oxidase I (COI) metabarcoding datasets (DnoisE) [20]. As this combination of methods has not been thoroughly investigated using 16S rRNA datasets, direct empirical comparisons for different microbial systems are necessary [21, 22]. Here, processing sequences using both methods allowed us to determine if program choice significantly impacts microbial analysis in ascidian hosts. We hypothesized there would be no significant differences in microbial alpha‐ or beta‐diversity results between OTU and ASV datasets. Additionally, we hypothesized that bacterial diversity and composition would be significantly different among body regions and across color morphs.

## 2. Materials and Methods

### 2.1. Ascidian and Seawater Collection

**Figure 1:**
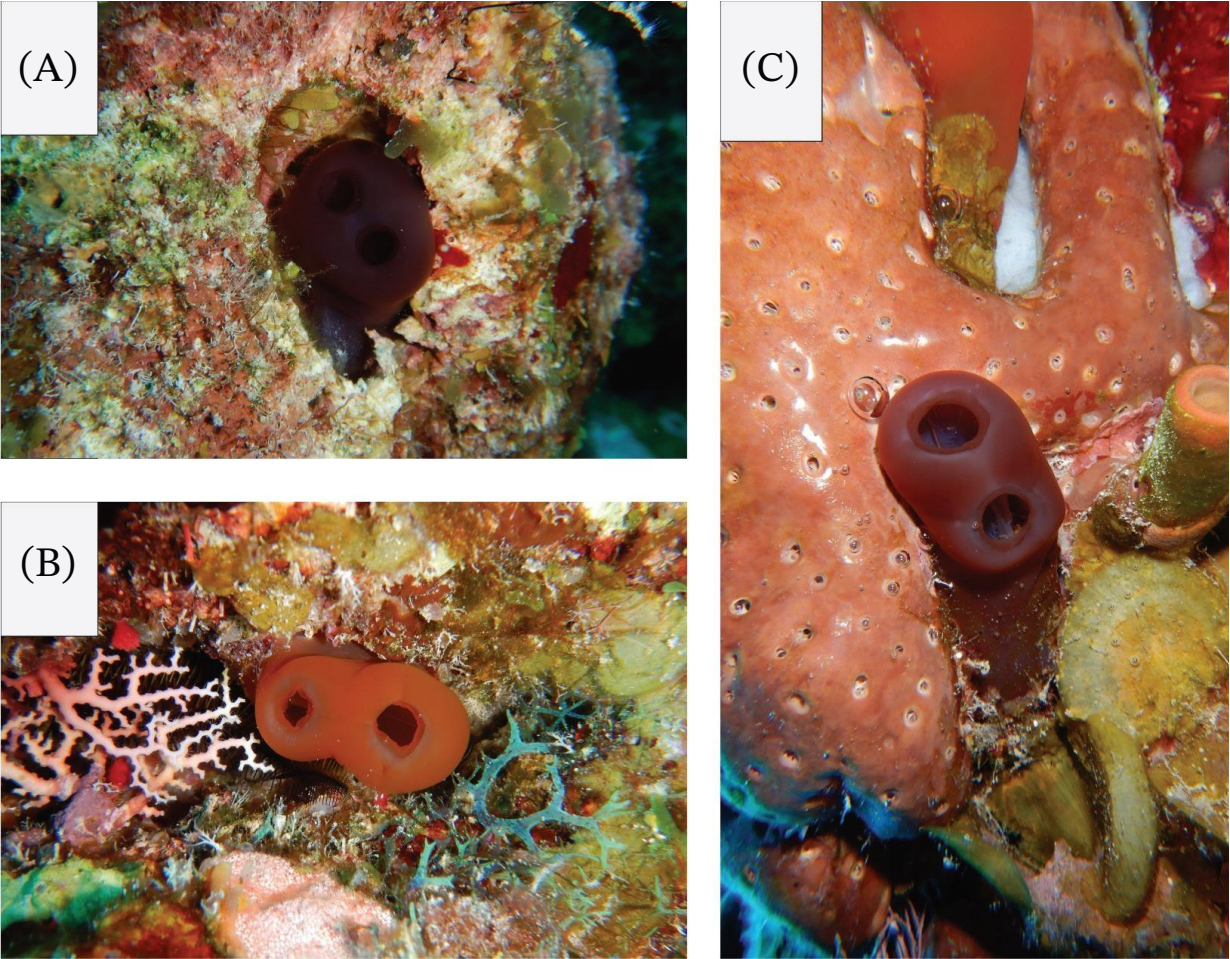
(A) Purple, (B) orange, and (C) intermediate individuals of *Rhopalaea abdominalis* in situ at Belizean reef sites.

Individuals of the solitary aplousobranch *R. abdominalis* were collected from four Belizean reef sites with max depths ranging from 10 to 21 m (Table 1) on July 7 and 8, 2023. Individuals were classified into three color morphs based on tunic coloration: purple (Figure 1A), orange (Figure 1B), or intermediate (Figure 1C), which appeared in shades of pink. A total of four purple, five orange, and four intermediate individuals were collected, held in ambient seawater with menthol crystals for 3 h to relax the ascidian tissues, and dissected at Carrie Bow Cay Field Station to isolate samples of the tunic, branchial sac, and gut tissue with contents. Tunic tissue was sampled from the central body (midway between siphons and basal regions), and gut tissue was sampled from intestines after the stomach region. All tissue samples were stored in 95% ethanol, with daily changes until July 12, after which samples were placed in 100% ethanol at −20°C for long‐term storage. At two of the collection sites (Table 1), three replicates of 500 mL of ambient seawater were collected and filtered through a sterile 0.2‐*μ*m filter with a Nalgene vacuum filtration system to collect bacterioplankton samples. Filters were stored in RNAlater at −20°C for long‐term storage.

**Table 1:** Site, GPS coordinates and max depth in feet, and the date that samples (ID) were collected in Belize during 2023. Color morph of each ascidian sample is indicated as either purple (P), orange (O), or intermediate (I). Cytochrome Oxidase I (COI) phylogenetic clade of each ascidian sample is also shown (see Figure 2, “n.a.” = not available). Ambient seawater samples are indicated in the color column with “—”.

Site	GPS/max depth	Date	ID	Color	COI
Fore‐reef at Carrie Bow Cay	16.802395; −88.078636/66 ft	July 7	7J23‐1‐2A	P	Clade I
			7J23‐1‐2B	P	n.a.
			7J23‐1‐2C	P	Clade I
			7J23‐1‐3A	O	Clade II
			7J23‐1‐3B	O	Clade II

Long‐reef at Carrie Bow Cay	16.777956; −88.079407/33 ft	July 7	7J23‐2‐7	O	Clade II
			7J23‐2‐AW	—	—
			7J23‐2‐BW	—	—
			7J23‐2‐CW	—	—

Tobacco Caye Deep	16.896936; −88.056148/70 ft	July 8	8J23‐1‐3A	O	Clade I
			8J23‐1‐3B	O	Clade II
			8J23‐1‐6A	I	Clade I
			8J23‐1‐6B	I	Clade II
			8J23‐1‐AW	—	—
			8J23‐1‐BW	—	—
			8J23‐1‐CW	—	—

Tobacco Caye Shallow	16.897316; −88.058959/36 ft	July 8	8J23‐2‐2A	I	Clade I
			8J23‐2‐2B	I	Clade I
			8J23‐2‐3	P	Clade I

### 2.2. Ascidian Phylogenetic Analyses

A portion of the dissected branchial sac tissue from each *R. abdominalis* individual was used for DNA extraction using the DNeasy Blood and Tissue Kit (QIAGEN). In addition to following the manufacturer′s instructions, 20 *μ*L RNase (10 mg/mL) was added to each sample after cell lysis in a Thermomixer (56°C, 350 rpm). The universal primers LCO1490/HCO2198 [23] were used to amplify a region of the COI gene. Polymerase chain reactions (PCRs) consisted of 0.5 *μ*L of the forward and reverse primer set, 11 *μ*L of PCR water, 12.5 *μ*L of MyTaq HS Red Mix, and 0.5 *μ*L of DNA. Reactions were performed at 95°C for 1 min; 35 cycles of 95°C for 15 s, 45°C for 15 s, and 72°C for 10 s; 72°C for 1 min; and a holding temperature of 4°C on an Eppendorf Mastercycler Nexus X2.

Successful amplification was verified by gel electrophoresis, and samples were purified using the QIAquick PCR Purification Kit (QIAGEN). Resulting products were used in sequencing reactions consisting of 5.5 *μ*L PCR water, 1.0 *μ*L BigDye Terminator v.3, 2.0 *μ*L BigDye Buffer (×5 concentration), 0.5 *μ*L of the forward or reverse primers, and 1.0 *μ*L of purified PCR product on an Eppendorf Mastercycler Nexus X2. Reactions were performed at 96°C for 1 min; 25 cycles of 50°C for 5 s, 60°C for 4 min, and 96°C for 10 s; 50°C for 5 s; 60°C for 4 min; and a holding temperature of 10°C. Sequencing was completed using the BigDye XTerminator Purification Kit (Applied Biosystems) and LCO1490/HCO2198 primers on an Applied Biosystems 3500 genetic analyzer at the UNCW Center for Marine Science. Sequences were aligned in Geneious (v. R11; Biomatters, Auckland, New Zealand), trimmed to 500 bp, and dereplicated into 11 haplotypes across 12 samples. All acquired sequences were deposited in GenBank (accession numbers: PX453699–PX453710).

A phylogenetic tree was constructed for the acquired COI sequences using the maximum‐likelihood (ML) and neighbor‐joining (NJ) methods in MEGA v.12 [24]. The best evolutionary model was determined to be Tamura–Nei with invariant sites, and the trees were constructed using this model and 1000 bootstrap replicates. A COI sequence for the solitary phlebobranch *Phallusia nigra* (Acc. MT637958.1) was included as an outgroup, and all previously available COI sequences for *R. abdominalis* on GenBank (Acc. MH258881‐3) were included in the phylogenetic tree.

### 2.3. Characterization of Ascidian Microbiome

A portion of the dissected tunic, branchial sac, and gut samples (ca. 8 mm^3^) from each *R. abdominalis* individual and a piece of each filter containing seawater bacterioplankton samples were used for DNA extractions with the DNeasy Blood and Tissue Kit (QIAGEN). DNA extraction kit negative controls, often recommended for low biomass samples, were not included in this study as ascidians commonly host dense microbial cell counts (2.6–8.8 × 10^9^ cells/cm^3^) [25]. Previous studies have reported little to no kit contamination at these cell densities (DNeasy Blood and Tissue Kit) [26], and sequence contaminants previously detected did not match common OTUs/ASVs in our dataset (see below and Table S2) [26], indicating minimal risk of kit contamination. Quality of DNA extractions was quantified with a NanoDrop One Spectrophotometer, and the V4 region of the 16S rRNA gene was amplified using the 515f/806r primers [27]. PCR reactions consisted of 0.5 *μ*L of the forward and reverse primers, 11 *μ*L of PCR water, 12.5 *μ*L of MyTaq HS Red Mix, and 0.5 *μ*L of DNA. Reactions were conducted on an Eppendorf Mastercycler Nexus X2 under the following conditions: 95°C for 2 min; 35 cycles of 95°C for 15 s, 50°C for 15 s, and 72°C for 20 s; 72°C for 2 min; and a holding temperature of 10°C. Results were visualized with gel electrophoresis, and 50 *μ*L of quality DNA extracts were sent to Zymo Research Corporation (Irvine, California, United States) for next‐generation sequencing (Illumina NextSeq; paired‐end 2 × 300 bp, which yielded an average of 305,577 raw sequences per sample) of the V4 region of the 16S rRNA gene.

Returned sequences were processed in two programs: the mothur software package (v.1.43.0) [16], which produced OTU counts, and QIIME 2 (v.2024.2) [28] with DADA2 [17], which resulted in ASV counts. For mothur, the pipeline described in Erwin et al. [29] was followed, and sequences were aligned to the SILVA taxonomy database (v132.V4; full bioinformatics pipeline details in Table S1) using classify. seqs (naïve Bayesian classifier) [30]. Rare OTUs (occurring one time in the entire dataset) were removed, and sequences were subsampled to the lowest sampling depth across all samples (*n* = 29,142). The full bioinformatics pipeline followed in QIIME 2 is detailed in Table S2. The default parameters were used for DADA2, including chimera removal and taxonomy assignment to the SILVA taxonomy database using classify‐sklearn (naïve Bayesian classifier) [31]. To maintain consistency with the mothur outputs, ASV sequences were subsampled to *n* = 29,142.

One sample failed quality checks with the QIIME 2 pipeline but was retained in the mothur pipeline. This sample, “7Jul23 1‐2B‐T,” corresponded to a purple tunic sample and was excluded from both OTU and ASV datasets for all analyses to maintain consistency. Additionally, updated microbial taxonomic nomenclature [32] has been used throughout this manuscript, and raw 16S rRNA sequences have been deposited in NCBI SRA (PRJNA1339030). OTU and ASV tables with count data by sample, full taxonomy, and representative sequences are provided as File S1.

### 2.4. Microbiome Statistical Analyses

To test for significant differences in microbial Shannon′s *H* ^′^, richness, and Pielou′s evenness across samples, a Kruskal–Wallis test was performed in RStudio (v.2024.04.1 + 748; R Core Team) using the stats package, followed by post hoc Dunn tests for multiple comparisons using the FSA package [33] with Holm‐adjusted *p* values to test for pairwise significance between samples. These tests were performed for the factors body region (branchial sac, gut, tunic, and seawater) and color morph (purple, orange, intermediate, and seawater) on both OTU and ASV datasets. Data were visualized as boxplots created with the ggplot2 package [34] in RStudio. To further test for significant differences in microbial alpha‐diversity metrics by sequence processing pipeline (OTU vs. ASV) and host COI clade (Clade I vs. Clade II), paired and two‐sample *t*‐tests were performed, respectively.

To determine significant differences in microbiome composition among samples, the Bray–Curtis dissimilarity was calculated. Permutational analyses of variance (PERMANOVAs) were completed using the adonis2 command from the package vegan [35] in RStudio for the factors body region, color morph, and an interaction term (body region × color morph). Pairwise PERMANOVAs were conducted using the adonis_pairwise command from the package metagMisc [36] in RStudio for body region and color morph, with beta dispersion also calculated. PERMANOVAs and pairwise PERMANOVAs were completed separately for OTU and ASV datasets. Results were visualized in a nonmetric multidimensional scaling (nMDS) plot for both OTU and ASV datasets separately, using the ggplot2 package in RStudio. To further test for significant differences in beta‐diversity by host COI clade and sample location, additional PERMANOVAs were calculated, and nMDS plots were constructed separately for both OTU and ASV datasets. Finally, congruence between beta‐diversity calculations (Bray–Curtis dissimilarity) based on OTU and ASV datasets was assessed with Mantel tests conducted in R (vegan package, mantel function).

OTU‐ and ASV‐level analyses were conducted for fine‐scale investigations of core and differentially abundant taxa among body regions and color morphs. Core OTUs and ASVs (strictly defined as taxa detected in all samples) were identified for each color morph (purple, orange, and intermediate) and for each body region within color morph (branchial sac, gut, and tunic). To identify OTUs and ASVs that exhibited significantly different relative abundance between sample types (body region and color morph), a multiple linear regression was run in MicrobiomeAnalyst 2.0 [37] using the linear model option with FDR‐adjusted *p* values.

## 3. Results

### 3.1. Ascidian Phylogenetic Analyses

**Figure 2:**
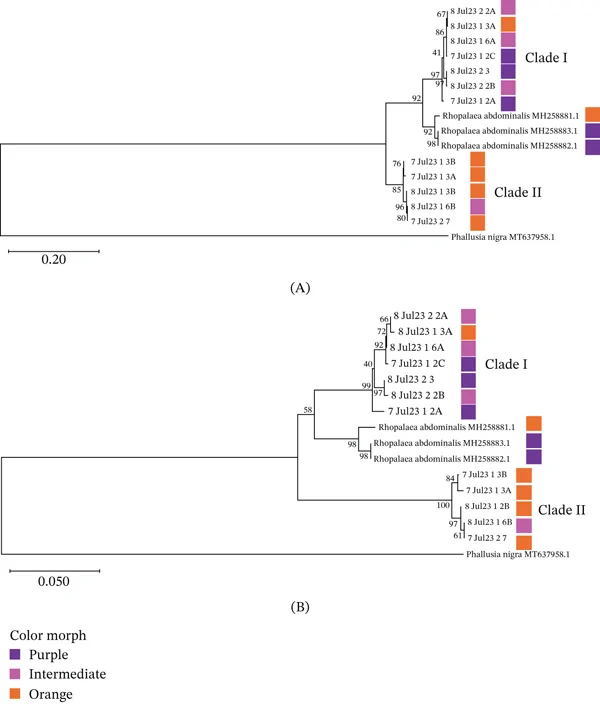
(A) Maximum‐likelihood and (B) neighbor‐joining phylogenetic trees of ascidian COI sequences inferred using the Tamura–Nei model, percentage of replicate trees (out of 1000 replicates) in which samples clustered together is indicated next to the branches. The color morph of each sample is indicated by color (purple, intermediate [pink], or orange) to the right of the sample name.

COI sequences were generated for 12 of 13 ascidians, with all color morphs represented (three purple, five orange, and four intermediate). These *R. abdominalis* samples exhibited high genetic diversity, with 12 COI sequences representing 11 distinct haplotypes that had an overall mean distance of 8%. Phylogenetic analyses using ML (Figure 2A) and NJ (Figure 2B) methods revealed three well‐supported monophyletic clades of *R. abdominalis*: two consisting of haplotypes generated herein (Clade I and Clade II; Figure 2) and a third formed by three publicly available COI sequences (one orange: acc. MH258881; two purple: acc. MH258882‐3) from individuals sampled in the Bahamas. The within‐group mean distance (number of base substitutions per site from averaging over all sequence pairs within each group, using the Tamura–Nei model) for Clade I, Clade II, and the Bahamas group was all 1%. The mean distance between groups (number of base substitutions per site from averaging over all sequence pairs between groups, using the Tamura–Nei model) between Clade I and Clade II was 14.3%, between Clade I and the Bahamas group was 7.32%, and between Clade II and the Bahamas group was 12.71%. *Rhopalaea abdominalis* haplotypes did not clearly cluster by color morph, with purple colonies clustered in Clade I but orange and intermediate colonies present in both Clade I and Clade II (Figure 2).

### 3.2. Characterization of Ascidian Microbiome

Sequencing yielded over 10 million raw reads from 38 ascidian and seawater samples. Following the processing of 16S rRNA sequences in mothur, a total of 1,107,396 high‐quality sequences representing 13,042 OTUs were identified across nine tunic (two purple, four orange, and three intermediate), 13 branchial sac (four purple, five orange, and four intermediate), 10 gut (three purple, three orange, and four intermediate), and six ambient seawater samples (Table S3). Similarly, QIIME 2 analysis resulted in 1,107,396 high‐quality sequences representing 13,386 ASVs across the same samples. Subsampling removed 89.01% of sequence reads but only 25.50% of OTUs and 18.37% of ASVs. When directly comparing the representative sequences for OTUs and ASVs, there were 6269 OTUs that were exact matches to ASVs (48.068%) and 6244 ASVs that were exact matches to OTUs (46.646% of total ASVs). These exact matches accounted for 97.705% of total OTU sequence counts but only 73.330% of total ASV sequence counts.

**Figure 3:**
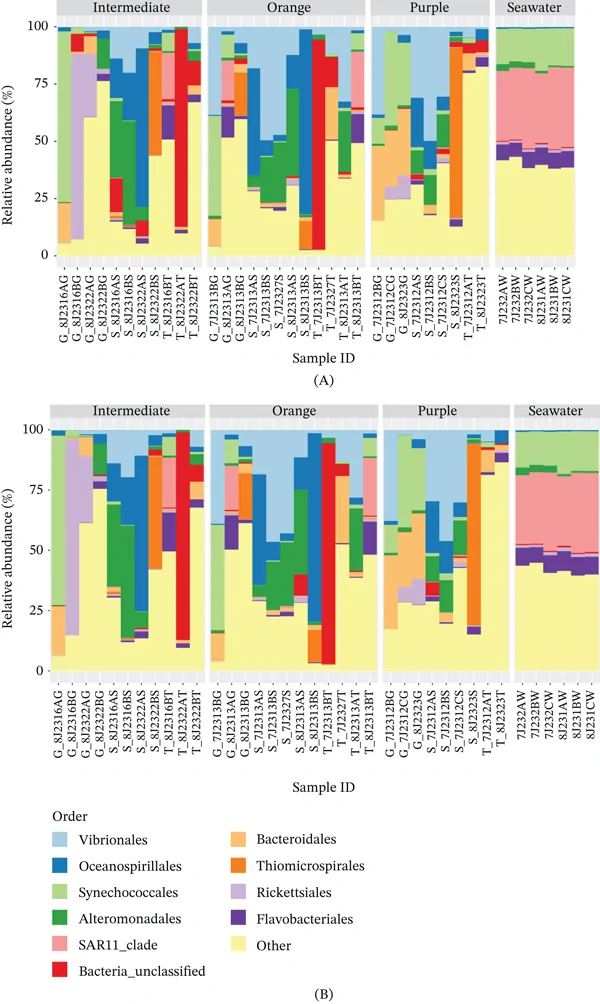
Stacked bar graphs of order‐level bacterial relative abundance within each sample for (A) OTU and (B) ASV datasets. The Top 10 most abundant orders within the dataset are shown, with all other orders represented within the “Other” category. Samples are grouped by color morph, and body region is indicated by the first letter of the corresponding “Sample ID”: branchial sac (S), gut (G), and tunic (T).

**Figure 4:**
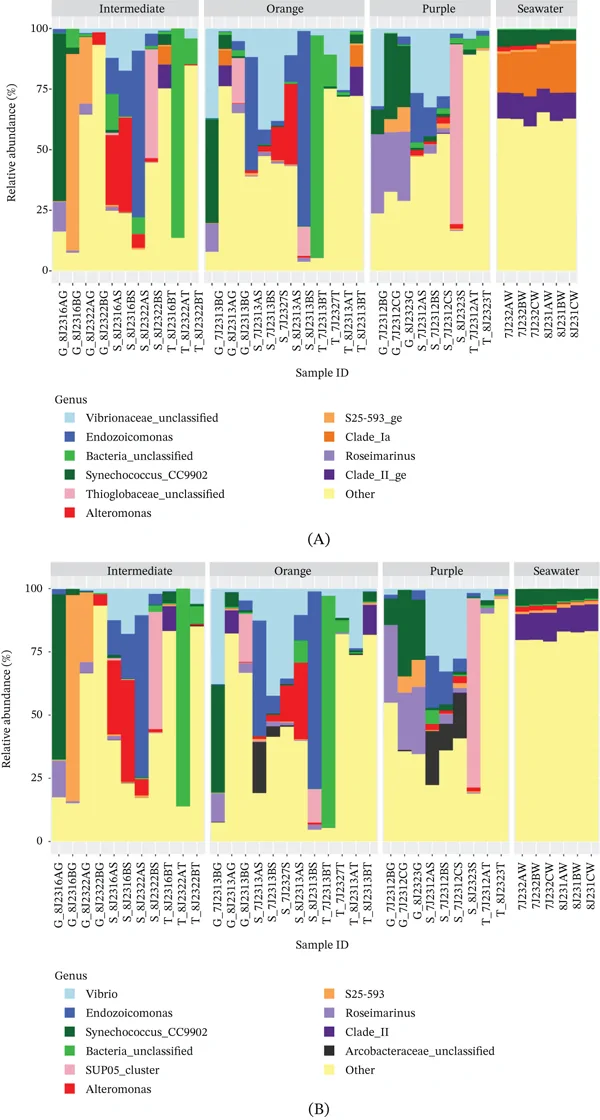
Stacked bar graphs of genus‐level bacterial relative abundance within each sample for (A) OTU and (B) ASV datasets. The Top 10 most abundant genera within the dataset are shown, with all other genera represented within the “Other” category. Samples are grouped by color morph, and body region is indicated by the first letter of the corresponding “Sample ID”: branchial sac (S), gut (G), and tunic (T).

Of the total OTUs in the dataset, 95.18% were bacteria and 4.82% were archaea (Figure S1A), whereas the ASV dataset consisted of 95.41% bacteria and 4.59% archaea (Figure S1B). The most abundant of the archaeal taxa in both the OTU and ASV datasets were members of the classes Nitrososphaeria and Thermoplasmata. The distribution of phyla Pseudomonadota, Bacteroidota, Cyanobacteria, unclassified bacteria, and Planctomycetota was congruent between the OTU (Figure S2A) and ASV (Figure S2B) datasets, both indicating that samples “8Jul23 2‐2A‐T” and “7Jul23 1‐3B‐T” were dominated by unclassified bacteria, which were found at far lower abundances in other samples. At the level of class, trends between OTU (Figure S3A) and ASV (Figure S3B) datasets were largely similar for Gammaproteobacteria, Alphaproteobacteria, Bacteroidia, Oxyphotobacteria (“Cyanobacteria” in ASV dataset), unclassified bacteria, Campylobacteria, and Fusobacteriia. The identity of the most abundant orders (Figure 3) and families (Figure S4) across all samples was identical between the OTU and ASV datasets, and their distributions were highly congruent (Figure 3; Figure S4). At the level of genus, distributions of most taxa were highly similar between OTU and ASV datasets, with slight differences including *Clade Ia* ranked 13^th^ (within “Other”) in the ASV dataset, whereas it was within the Top 10 in the OTU dataset (Figure 4A) and *Arcobacteraceae* being within the Top 10 genera in the ASV dataset (Figure 4B).

### 3.3. Microbiome Statistical Analyses

#### 3.3.1. Alpha‐Diversity

**Figure 5:**
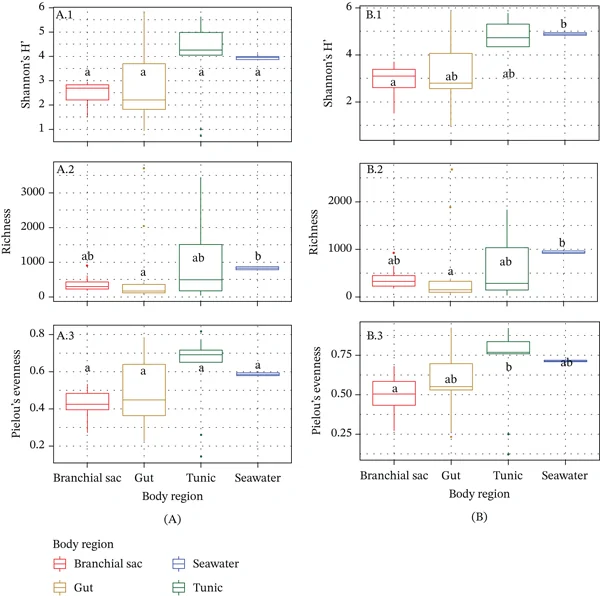
Boxplots built from (A) OTU and (B) ASV datasets displaying alpha‐diversity metrics (1) Shannon′s *H* ^′^ diversity, (2) richness, and (3) evenness by body regions (branchial sac = red, gut = brown, tunic = green, and seawater = blue). Significant differences between sample types, as calculated by a pairwise Dunn test with Holm‐adjusted *p* values, are indicated by lowercase letters.

Comparing body region across samples for OTU data indicated significant differences in microbial Shannon′s *H* ^′^ diversity (Kruskal–Wallis; *p* = 0.033; Figure 5A.1; Table 2) and evenness (*p* = 0.032; Figure 5A.3; Table 2). Following post hoc tests, however, the only significant pairwise comparison was that seawater was richer than gut samples (*p* = 0.037; Table 3). ASV data displayed significant differences in microbial Shannon′s *H* ^′^ diversity (*p* = 0.013; Figure 5B.1; Table 2), richness (*p* = 0.042; Figure 5B.2; Table 2), and evenness (*p* = 0.013; Figure 5B.3; Table 2) across samples by body region. Post hoc tests revealed significant results for microbial Shannon′s *H* ^′^ diversity and richness between ascidian samples and seawater (Table 3) and that the branchial sac samples had a significantly lower evenness than tunic samples (*p* = 0.019; Table 3).

**Table 2:** Chi‐squared (*X* ^2^) and *p* values (*p*) of the Kruskal–Wallis rank sum tests comparing body region (BR) and color morph (CM) across ascidian samples and seawater for Shannon′s *H* ^′^ diversity (*H* ^′^), richness, and evenness by OTU and ASV datasets. Significant values are in bold.

	OTU						ASV					
	*H* ^′^		Richness		Evenness		*H* ^′^		Richness		Evenness	
	*X* ^2^	*p*	*X* ^2^	*p*	*X* ^2^	*p*	*X* ^2^	*p*	*X* ^2^	*p*	*X* ^2^	*p*
BR	8.72	**0.033**	7.61	0.055	8.81	**0.032**	10.79	**0.013**	8.18	**0.042**	10.77	**0.013**
CM	3.64	0.303	9.44	**0.024**	2.68	0.444	9.21	**0.027**	11.20	**0.011**	3.27	0.352

**Table 3:** Unadjusted *p* values (*p*) and Holm‐adjusted *p* values (*p*‐adj) as calculated by pairwise Dunn test comparing samples by body region (BR) (branchial sac [S], gut [G], tunic [T], and seawater [SW]) and color morph (CM) (purple [P], orange [O], intermediate [I], and seawater [SW]) for Shannon′s *H* ^′^ diversity (*H* ^′^), richness, and evenness by OTU and ASV datasets. Significant values following post hoc tests are in bold.

Sample	Comparison	OTU						ASV					
		*H* ^′^		Richness		Evenness		*H* ^′^		Richness		Evenness	
		*p*	*p*‐adj	*p*	*p*‐adj	*p*	*p*‐adj	*p*	*p*‐adj	*p*	*p*‐adj	*p*	*p*‐adj
BR	S × G	0.796	1.000	0.295	0.590	0.415	0.831	0.527	0.527	0.216	0.648	0.189	0.567
	S × SW	0.042	0.211	0.049	0.244	0.031	0.157	0.003	**0.016**	0.053	0.265	0.017	0.087
	G × SW	0.083	0.250	0.006	**0.037**	0.163	0.490	0.018	0.092	0.004	**0.026**	0.229	0.458
	S × T	0.017	0.100	0.733	0.733	0.010	0.060	0.046	0.186	0.973	0.973	0.003	**0.019**
	G × T	0.043	0.173	0.201	0.602	0.092	0.367	0.193	0.579	0.271	0.542	0.114	0.457
	SW × T	0.947	0.947	0.118	0.470	0.917	0.917	0.240	0.479	0.066	0.263	0.842	0.842

CM	I × O	0.256	1.000	0.078	0.310	0.487	1.000	0.122	0.367	0.044	0.175	0.383	1.000
	I × P	0.711	0.711	0.841	0.841	0.525	1.000	0.578	0.578	0.833	0.833	0.318	1.000
	O × P	0.486	0.971	0.143	0.428	0.991	0.991	0.370	0.741	0.090	0.270	0.847	0.847
	I × SW	0.076	0.455	0.007	**0.042**	0.102	0.611	0.004	**0.023**	0.004	**0.023**	0.075	0.449
	O × SW	0.393	1.000	0.205	0.410	0.280	1.000	0.099	0.396	0.213	0.426	0.280	1.000
	P × SW	0.163	0.816	0.015	0.076	0.301	1.000	0.021	0.103	0.009	**0.047**	0.388	0.776

**Figure 6:**
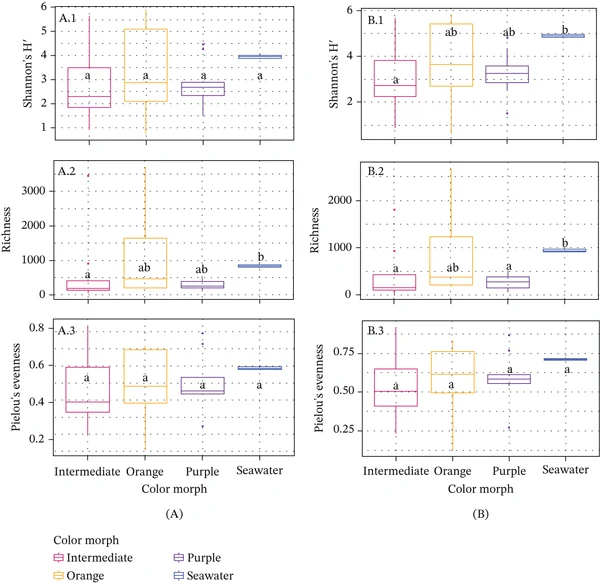
Boxplots built from (A) OTU and (B) ASV datasets displaying alpha‐diversity metrics (1) Shannon′s *H* ^′^ diversity, (2) richness, and (3) evenness by color morphs (intermediate = pink, orange = orange, purple = purple, and seawater = blue). Significant differences between sample types, as calculated by a pairwise Dunn test with Holm‐adjusted *p* values, are indicated by lowercase letters.

When comparing color morph across samples for OTU data, the Kruskal–Wallis rank sum tests indicated no significant differences in microbial Shannon′s *H* ^′^ diversity (Figure 6A.1) or Pielou′s evenness (Figure 6A.3; Table 2), whereas there was a significant difference in richness (*p* = 0.024; Figure 6A.2). Post hoc tests revealed that this significant difference in microbial richness was driven by seawater samples having a higher richness than ascidian samples (Table 3). For ASV data comparing color morph across samples, microbial Shannon′s *H* ^′^ diversity (*p* = 0.027; Figure 6B.1) and richness (*p* = 0.011; Figure 6B.2) were significantly different, whereas evenness was nonsignificant (Figure 6B.3; Table 2). Again, post hoc tests revealed that all significant pairwise differences were between ascidian samples and seawater, with seawater microbial communities being significantly more diverse and richer than ascidian samples (Table 3).

Microbial alpha‐diversity metrics (Shannon′s *H* ^′^ diversity, richness, and Pielou′s evenness) for all samples based on OTU and ASV datasets are reported in Table S3. A paired *t*‐test indicated no significant difference in richness values between OTU and ASV datasets. However, the ASV‐based processing method resulted in significantly higher diversity and evenness values (Table S4). No significant differences in alpha‐diversity metrics were observed between host COI clades based on OTU or ASV datasets (Table S5).

#### 3.3.2. Beta‐Diversity

**Figure 7:**
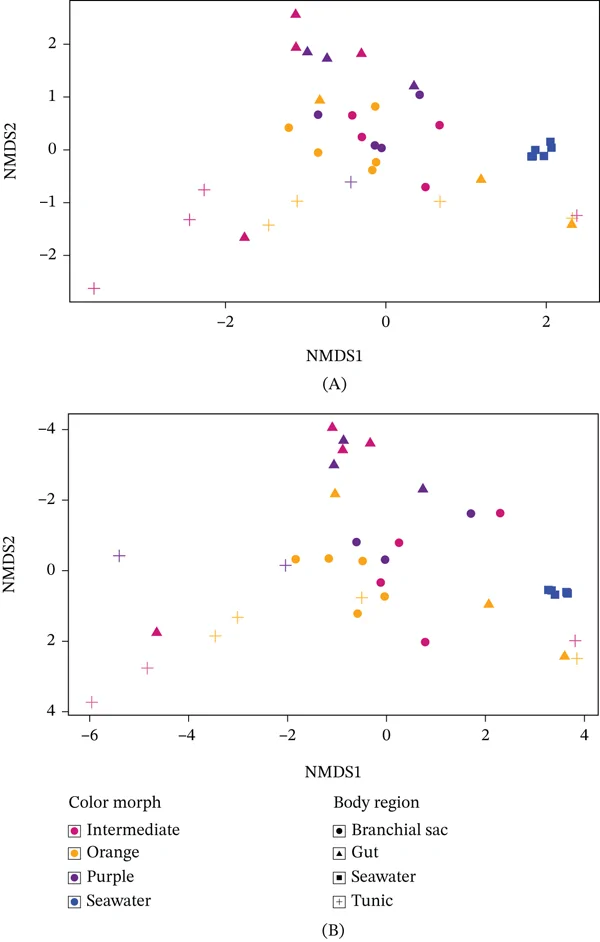
Nonmetric multidimensional scaling plots displaying data from (A) OTU (stress = 0.108) and (B) ASV (stress = 0.112) datasets. Body region is denoted by shape: branchial sac (circle), gut (triangle), seawater (square), and tunic (cross); color morph is indicated by color: intermediate (pink), orange (orange), purple (purple), and seawater (blue).

Microbiome composition was significantly different (*p* = 0.001) among samples based on body region, color morph, and their interaction for both OTU and ASV datasets (Table 4). Pairwise PERMANOVAs by body region indicated that all comparisons were significantly different for both OTU and ASV datasets, but beta dispersions indicated a homogenous (nonsignificant) dispersion between branchial sac and gut (OTU, *p* = 0.283; ASV, *p* = 0.439) and between gut and tunic (OTU, *p* = 0.373; ASV, *p* = 0.437) samples (Table 5). Pairwise PERMANOVAs by color morph indicated that only comparisons between ascidian samples and seawater samples were significantly different for both OTU (*p* = 0.002 for all) and ASV (*p* = 0.002 for all) datasets, with beta dispersion results following the same trend (Table 5). Accordingly, nMDS plots based on OTU (stress = 0.108; Figure 7A) and ASV (stress = 0.110; Figure 7B) datasets illustrated a lack of clustering of ascidian samples by color morph.

**Table 4:** *R* ^2^, *F*‐statistic (*F*), and *p* values (*p*) from PERMANOVA analyses for body region (BR), color morph (CM), and their interaction by OTU and ASV datasets. Significant values are in bold.

	OTU			ASV		
	*R* ^2^	*F*	*p*	*R* ^2^	*F*	*p*
BR	0.336	5.742	**0.001**	0.318	5.290	**0.001**
CM	0.220	3.190	**0.001**	0.214	3.087	**0.001**
BR × CM	0.483	2.906	**0.001**	0.475	2.817	**0.001**

**Table 5:** *R* ^2^, *F*‐statistic (*F*), *p* value (*p*), FDR‐adjusted *p* values (*p*‐adj) from PERMANOVA and beta dispersion analyses for body region (BR) (branchial sac [S], gut [G], tunic [T], and seawater [SW]) and color morph (CM) (purple [P], orange [O], intermediate [I], and seawater [SW]) by OTU and ASV datasets. Significant values following post hoc are in bold.

Sample	Comparison	OTU							ASV						
		PERMANOVA				Beta dispersion			PERMANOVA				Beta dispersion		
		*R* ^2^	*F*	*p*	*p*‐adj	*F*	*p*	*p*‐adj	*R* ^2^	*F*	*p*	*p*‐adj	*F*	*p*	*p*‐adj
BR	S × G	0.191	4.968	0.001	**0.001**	1.414	0.236	0.283	0.178	4.547	0.001	**0.001**	0.707	0.439	0.439
	S × SW	0.460	14.480	0.001	**0.001**	55.707	0.001	**0.002**	0.414	12.029	0.001	**0.001**	83.901	0.001	**0.002**
	G × SW	0.366	8.089	0.001	**0.001**	42.188	0.001	**0.002**	0.361	7.900	0.001	**0.001**	48.582	0.001	**0.002**
	S × T	0.173	4.200	0.001	**0.001**	8.952	0.006	**0.009**	0.160	3.822	0.001	**0.001**	7.434	0.005	**0.008**
	G × T	0.124	2.400	0.002	**0.002**	0.872	0.373	0.373	0.122	2.369	0.003	**0.003**	1.010	0.364	0.437
	SW × T	0.315	5.965	0.001	**0.001**	2408.257	0.001	**0.002**	0.304	5.688	0.001	**0.001**	1546.496	0.001	**0.002**

CM	I × O	0.042	0.921	0.563	0.563	2.042	0.169	0.203	0.049	1.090	0.324	0.324	1.305	0.271	0.325
	I × P	0.063	1.201	0.203	0.305	2.767	0.124	0.186	0.060	1.153	0.235	0.282	2.288	0.156	0.234
	O × P	0.057	1.150	0.279	0.335	0.101	0.721	0.721	0.064	1.291	0.156	0.234	0.463	0.531	0.531
	I × SW	0.292	6.187	0.001	**0.002**	386.542	0.001	**0.002**	0.286	6.002	0.001	**0.002**	420.312	0.001	**0.002**
	O × SW	0.314	7.328	0.001	**0.002**	160.306	0.001	**0.002**	0.288	6.469	0.001	**0.002**	366.015	0.001	**0.002**
	P × SW	0.400	8.665	0.001	**0.002**	131.469	0.001	**0.002**	0.388	8.239	0.001	**0.002**	152.536	0.001	**0.002**

In addition to these analyses, PERMANOVAs indicated no significant differences in microbial composition of ascidian samples by phylogenetic clade (I and II; Figure 2) or collection location for OTU and ASV datasets (Tables S6 and S7). Accordingly, a lack of clustering of ascidian samples by clade and location is apparent in nMDS plots based on OTU and ASV datasets (Figures S5 and S6). Finally, Mantel tests revealed strong congruence and significant correlation between dissimilarity values calculated with OTU and ASV datasets (*r* = 0.9658, *p* < 0.001).

#### 3.3.3. Core OTUs and ASVs

**Figure 8:**
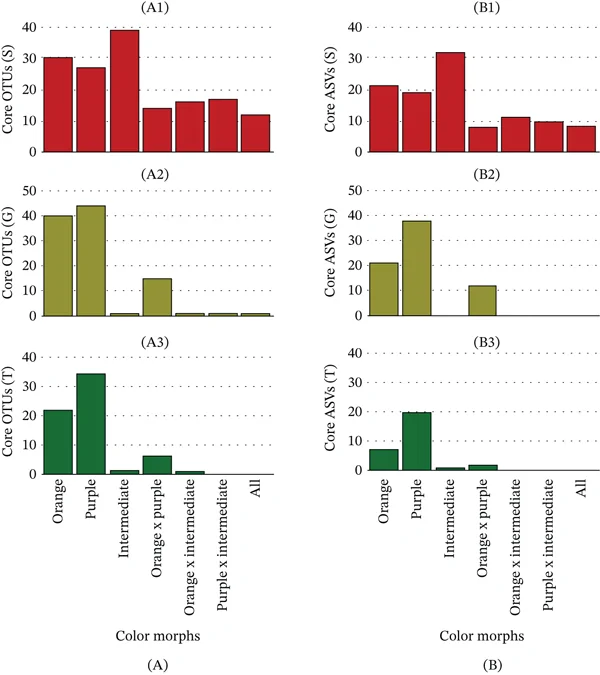
Number of core (A) OTUs and (B) ASVs for each body region by color morph. Body region is denoted by color: (A.1,B.1) branchial sac (red), (A.2,B.2) gut (brown), and (A.3,B.3) tunic (green).

Patterns of core microbial taxa across body regions and color morphs were congruent between OTU and ASV datasets, with a consistently greater number of core OTUs than core ASVs in *R. abdominalis* (Figure 8; Table S8). Microbial communities in tunic samples had the fewest core OTUs and ASVs on average compared with branchial sac and gut body regions within each color morph (Figure 8; Table S8). When combinations of color morphs were considered, the branchial sac had the most core OTUs (0.113% of total OTUs) and ASVs (0.069% of total ASVs) on average (Figure 8; Table S8).

Core OTUs belonging to the family Vibrionaceae and the genera *Endozoicomonas*, *Alteromonas*, *Ferrimonas*, and *Pseudoalteromonas* were conserved in the branchial sac of orange, purple, and intermediate color morphs (Table S9). These five taxa accounted for 0.038% of total OTUs but 12.003% of total sequence counts. A similar trend was observed for core ASVs in the branchial sac, where members of the genera *Endozoicomonas*, *Alteromonas*, *Vibrio*, *Pseudoalteromonas*, and *Ferrimonas* were core across all color morphs (Table S10), but these taxa accounted for 0.037% of total ASVs and only 11.184% of total sequence counts. The only core OTU in the gut of all color morphs was a member of the family Vibrionaceae, and no OTUs were core to all tunic samples across color morphs (Table S9). No ASVs were core to the gut or tunic of all color morphs (Table S10).

#### 3.3.4. Differentially Abundant OTUs and ASVs

Differential analyses of microbial taxa across body regions and color morphs were also congruent between OTU and ASV datasets. An average of 23 ± 8 OTUs (0.176% of total OTUs) were significantly different among *R. abdominalis* body regions, whereas an average of 501 ± 98 OTUs (3.841% of total OTUs) were significantly different between *R. abdominalis* samples and seawater (Table 6). Similarly, an average of 26 ± 6 ASVs (0.194% of total ASVs) were significantly different between comparisons of *R. abdominalis* body regions, and an average of 738 ± 65 ASVs (5.513% of total ASVs) were significantly different between *R. abdominalis* samples and seawater (Table 6). Comparisons among color morphs found an average of < 1 OTU (0.003%) and 2 ASVs (0.015%) that were significantly different, whereas 503 ± 49 OTUs (3.857%) and 726 ± 32 ASVs (5.424%) were significantly different between color morphs and seawater (Table 6).

**Table 6:** Number of significantly different OTUs and ASVs between sample type comparisons for body region (BR) (branchial sac [S], gut [G], tunic [T], and seawater [SW]) and color morph (CM) (purple [P], orange [O], intermediate [I], and seawater [SW]) using OTU and ASV datasets.

Sample	Comparison	OTU		ASV	
		# of OTUs	% out of 13,042	# of ASVs	% out of 13,386
BR	S × G	25	0.192	32	0.239
	S × T	30	0.230	29	0.217
	S × SW	611	4.685	792	5.917
	G × T	14	0.107	18	0.134
	G × SW	470	3.604	745	5.566
	T × SW	423	3.243	677	5.058

CM	I × O	0	0	4	0.030
	I × P	1	0.008	2	0.015
	I × SW	471	3.611	746	5.573
	O × P	0	0	1	0.007
	O × SW	559	4.286	757	5.655
	P × SW	479	3.673	677	5.058

**Figure 9:**
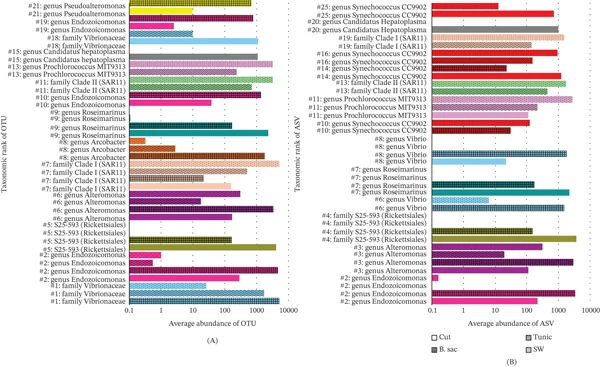
Average abundance of the Top 5 most abundant (A) OTUs and (B) ASVs that were significantly different between pairwise comparisons of body regions. Horizontal bars correspond to the average abundance of an OTU or ASV across all gut (solid), branchial sac (grid), tunic (zigzag), and ambient seawater (dots) samples. No horizontal bar indicates an average abundance of zero for that sample type. *y*‐axis displays the taxonomic rank of the OTU or ASV; *x*‐axis is displayed on a logarithmic scale.

Taxonomic identity of the five most abundant differential OTUs and ASVs by body region was fairly consistent across datasets, with these taxa consisting of 21.735% of all OTU sequence counts and 20.272% of all ASV sequence counts. The genera *Endozoicomonas* and *Alteromonas* were significantly more abundant in the branchial sac than in the gut or tunic. The genera *Roseimarinus* and *Candidatus* Hepatoplasma were significantly more abundant in the gut than in the tunic. Family S25‐593 (Rickettsiales) was significantly more abundant in the branchial sac and gut than in the tunic. Finally, members of the family Arcobacteraceae were significantly more abundant in the branchial sac than in the tunic (sixth most abundant and differential for ASVs; Figure 9A,B; Table S11).

**Figure 10:**
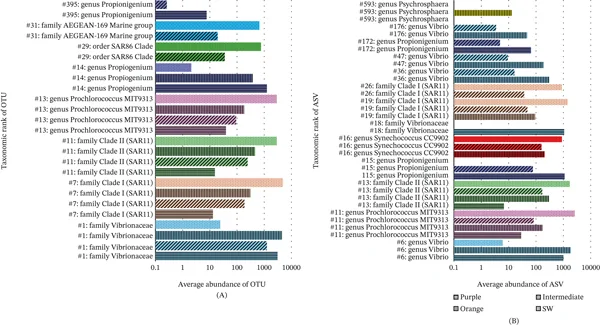
Average abundance of the top five most abundant (A) OTUs and (B) ASVs that were significantly different between pairwise comparisons of color morph. Horizontal bars correspond to the average abundance of an OTU or ASV across all purple (horizontal stripes), orange (vertical stripes), intermediate (diagonal stripes), and ambient seawater (dots) samples. No horizontal bar indicates an average abundance of zero for that sample type. *y*‐axis displays the taxonomic rank of the OTU or ASV; *x*‐axis is displayed on a logarithmic scale.

Taxonomic identity of the Top 5 most abundant differential OTUs and ASVs by color morph was less consistent, but all were rare taxa. For the OTU dataset, the purple color morph of *R. abdominalis* had significantly greater abundance of OTU395 (genus *Propionigenium*; accounting for 0.011% of total sequence counts) than the intermediate color morph (Figure 10A; Table S11). No OTUs were significantly different between orange and intermediate or purple color morphs. For the ASV dataset, members of the genera *Vibrio* (ASV36, 47, 176; 0.859% total sequence counts) and *Psychrosphaera* (ASV593; 0.015% total sequence counts) were significantly more abundant in the orange than the intermediate color morphs, *Propionigenium* (ASV15, 172; 1.141% total sequence counts) were significantly more abundant in the purple than the intermediate color morph, and *Psychrosphaera* (ASV593) were significantly more abundant in the orange than the purple color morph (Figure 10B; Table S11). The only case in which results were the same between the OTU and ASV datasets was for members of the genus *Propionigenium*, which were significantly more abundant in the purple than in the intermediate color morph (Figure 10A,B; Table S11).

## 4. Discussion

Analysis of the microbial communities associated with purple, orange, and intermediate color morphs of *R. abdominalis* indicated no significant differences in microbial alpha‐ or beta‐diversity metrics across color morphs but distinct microbiomes within the branchial sac, gut, and tunic body regions. Such compartmentalization of microbes by body region has also been documented in the solitary species *Styela plicata* [38], *Ascidia sydneiensis* [39], and, to a lesser extent, *Pyura vittata* [40]. Although this is the first microbiome characterization across color morphs for a solitary species, similarly conserved microbiomes have been documented between color morphs of the colonial *Polyclinum constellatum* [8] and *Cystodytes dellechiajei* [41], suggesting that host color variation can be independent of microbiome structure in both solitary and colonial species. These findings notably differ from other hosts, including amphipods, aphids, coral, and sea urchins, where bacterial symbionts may contribute to or inhibit pigmentation biosynthesis [42–45]. Additionally, results herein were consistent using both OTU‐ and ASV‐based processing methods, with no significant differences in alpha‐ or beta‐diversity and similar core microbiome profiles between programs. This strengthens the evidence that either processing technique can effectively elucidate trends in microbial community structure in a variety of systems, including terrestrial leaf‐litter [46], human milk [47], the intestine of Pacific whiteleg shrimp [48], human stomach [21], and now the branchial sac, gut, and tunic of a Belizean ascidian, in addition to ambient seawater.

Phylogenetic analyses of *R. abdominalis* revealed two clades unrelated to color morphs or microbiome structure. A similar lack of genetic differentiation in ascidians displaying multiple colorations has been reported for some colonial species [6, 8], whereas in other cases, color differences tracked with genotype and helped identify cryptic species [3–5]. Even in cases where host color does not correlate with genetic divergence or microbiome structure, different color morphs of the same species (e.g., blue and green morphs of *Cystodytes dellechiajei*) can have distinct chemical compositions and serve as a source of diverse marine natural products, such as sulfur‐containing pyridoacridines [49]. Therefore, future investigations into genetic and microbial correlates of ascidian color variation could benefit from establishing profiles for each color morph, as such information may prove valuable for pharmaceutical development.

Analysis of both OTU and ASV datasets identified the genera *Endozoicomonas*, *Alteromonas*, *Ferrimonas*, and *Pseudoalteromonas* as core to the branchial sac of individuals from all color morphs. Members of *Endozoicomonas* are facultative symbionts that feed on mucus within the branchial sacs of ascidian species [50], were recently identified as core members of the branchial sac of the solitary species *Styela plicata* [51], and represent ecologically important symbionts in other marine hosts, including corals (e.g., [52]). Symbiotic *Alteromonas* may assist their hosts in breaking down complex particulates like starches [53] that are brought into the branchial sac during filter‐feeding. In addition to being symbionts of ascidians, *Ferrimonas* are also core to the gut of black tiger shrimp (*Penaeus monodon*) [54] and colonize the gut of sea cucumbers (*Apostichopus japonicus*) [55]. *Pseudoalteromonas* have displayed strong antimicrobial activity when isolated from multiple ascidian species [56] and display vanadium‐accumulating and reducing properties in the intestine of *Ciona robusta* [57], which may be an adaptive response of the bacteria to prolonged exposure to harmful vanadium [58]. In the gut of *R. abdominalis*, there was one core OTU across all color morphs belonging to the family Vibrionaceae (100% match with *V. alginolyticus*; GenBank: MH169289.1), but no core ASVs. No core OTUs or ASVs were identified in the tunic across all color morphs, suggesting that the branchial sac harbors highly conserved microbial taxa of functional importance, compared with the more variable gut and tunic body regions.

Highly conserved microbes within the branchial sac of all color morphs were also among the most abundant and significantly different between body regions, with *Endozoicomonas* and *Alteromonas* being significantly more abundant in the branchial sac than in other body regions. Additionally, members of the family Arcobacteraceae were significantly more abundant in the branchial sac than in the tunic. Arcobacteraceae are found in diverse terrestrial and marine habitats and can utilize both heterotrophy and autotrophy [59], with recent interest in their ability to break down chemicals associated with plastic pollution, such as the hydrocarbon phenanthrene [60]. In marine invertebrate hosts that are fed nanoplastics [61] and microplastics [62], for example, members of Arcobacteraceae display significantly increased abundances. Other microbial taxa of interest include *Roseimarinus* and *Candidatus* Hepatoplasma, both of which were significantly more abundant in the gut than the tunic. *Roseimarinus* has been noted in high abundance in corals infected with stony coral tissue loss [63] and white plague [64] diseases and has been documented seasonally in the gut of sea urchins [65]. *Candidatus* Hepatoplasma is a dominant taxon among the microbiomes of multiple species of crab [66, 67], in the gut of sea urchins [65], and in the branchial sac of *Styela plicata* during warm seasons [51]. Finally, members of the family S25‐593 (Rickettsiales) were significantly more abundant in the branchial sac and gut than in the tunic and have previously been documented in the gut of *Ciona intestinalis* ([68]; GenBank: KF798911.1). Thus, microbial taxa that are significantly more abundant in the branchial sac of *R. abdominalis* include known ascidian symbionts and bacteria that may assist the host in the breakdown of pollutants. In contrast, those that are highly abundant in the gut may be transient microbes acquired via filter‐feeding that can colonize a diverse range of marine invertebrates.

OTU and ASV datasets showed slight variations in microbial taxa that were significantly different between color morphs, but all comprised bacteria commonly documented in marine habitats. In the OTU dataset, a member of the genus *Propionigenium* was more abundant in the purple morph than in the intermediate color morph. *Propionigenium* is a dominant taxon in the guts of both marine vertebrates and invertebrates [67] with no documented pigmentation. For the ASV dataset, *Propionigenium* was still significantly more abundant in the purple color morph, but *Vibrio* and *Psychrosphaera* were also significantly more abundant in the orange than in the intermediate color morphs. *Vibrio* can produce pigments that range from dark‐colored melanin [69] to red prodigiosin [70] and the diversely colored phenazine [71]. *Psychrosphaera*, however, are nonpigmented and have been isolated from deep‐sea sediment [72], seawater, and the gut of abalone [73]. Thus, some of the microbial taxa that were significantly more abundant in one ascidian color morph than another were capable of producing pigments, but spectrometry techniques would need to be implemented to determine if the concentration of these pigments also differed between host color morphs.

Analysis of ascidian microbiomes using OTU‐ and ASV‐based pipelines produced highly congruent results, including consistent alpha‐ and beta‐diversity patterns and similar core membership profiles. In theory, ASV‐based pipelines exhibit greater resolution than OTU‐based clustering, allowing differentiation of sequences by a single base pair, and analyses of mock datasets reported fewer erroneous sequences in ASV than in OTU datasets [22]. However, empirical studies analyzing sequence datasets using parallel pipelines have reported consistent overall conclusions across pipelines (e.g., 4 ASV‐based and 1 OTU‐based) [21], suggesting that general similarities in data trends surpass minor pipeline‐dependent differences. Our study results support this conclusion and further show remarkable similarity in taxonomic resolution between the analysis pipelines, yielding similar total richness values (13,042 OTUs and 13,386 ASVs) and no significant difference in average richness values across samples. Other study‐specific considerations may play a larger role in sequence processing pipeline choice, including comparisons of multiple datasets and data availability. For example, ASV‐based pipelines can more readily combine and compare ASVs from multiple datasets for analysis, due to the static nature of ASVs. In contrast, de novo OTUs are dependent on other sequences in the dataset and change with inclusion or removal of sequences from clustering algorithms [18]. Notably, the recently developed OptiFit algorithm [74] now allows new sequences to be clustered based on existing OTUs, facilitating the combination of datasets and confirming the continued relevance of OTU‐based pipelines. Ultimately, the existence of multiple sequence processing pipelines that produce congruent results with case‐specific advantages is a boon to microbiome science, supporting greater flexibility in study design while maintaining confidence in the detection of pipeline‐independent biological signals.

## 5. Conclusion

We demonstrated that microbiome diversity and structure were conserved across color morphs in the solitary ascidian *R. abdominalis*. However, the branchial sac, gut, and tunic body regions hosted distinct microbial communities. We also determined that the color variation observed in this species across Belizean reef populations was not indicative of speciation. This work contributes to growing evidence that symbiont communities correspond with host color morphs in ascidians when they represent genetically distinct lineages [13], whereas color morphs with no distinct phylogenetic lineage display no microbiome differences [8], as was the case herein. Continued characterization of ascidian microbiomes alongside phylogenetic analyses of hosts, particularly of the relatively understudied solitary species, is needed to clarify how symbiont communities are established and how they relate to biotic and abiotic parameters such as habitat, physiological activity, and competition.

## Author Contributions

Brenna Hutchings: conceptualization (equal), formal analysis (lead), and writing—original draft, review, and editing (lead). Susanna López‐Legentil: conceptualization (equal), formal analysis (supporting), funding acquisition (equal), writing—original draft, review, and editing (supporting). Lauren M. Stefaniak: funding acquisition (equal) and writing—review and editing (supporting). Marie L. Nydam: funding acquisition (equal) and writing—review and editing (supporting). Patrick M. Erwin: conceptualization (equal), formal analysis (supporting), funding acquisition (equal), and writing—original draft, review, and editing (supporting).

## Funding

This study was funded by the National Science Foundation, 10.13039/100000001, DEB‐2122475, and the Ruth D. Turner Foundation Scholarship in Marine Biology.

## Conflicts of Interest

The authors declare no conflicts of interest.

## Supporting information


**Supporting Information** Additional supporting information can be found online in the Supporting Information section. Additional supporting information can be found online. Additional figures include stacked bar graphs of relative abundance of microbial domains (Figure S1), phyla (Figure S2), classes (Figure S3), and families (Figure S4) and nonmetric multidimensional scaling plots of microbiomes coded by host phylogenetic clade (Figure S5) and collection location (Figure S6). Additional tables provide bioinformatics pipeline details (Tables S1 and S2), alpha diversity indices (Table S3), additional statistical comparisons of alpha diversity (Tables S4 and S5) and beta diversity (Tables S6 and S7), and the number (Table S8), taxonomic identity (Tables S9 and S10), and differential abundance (Table S11) of core OTUs and ASVs. File S1 includes OTU and ASV databases including count data by sample, full taxonomy, and representative sequences.

## Data Availability

The DNA sequence data that support the findings of this study are openly available in NCBI GenBank at https://www.ncbi.nlm.nih.gov/genbank/, references PX453699–PX453710, and NCBI SRA at https://www.ncbi.nlm.nih.gov/sra, reference number PRJNA1339030.
