# Supplementary material for: Microbial Communities Vary by Body Region but Remain Consistent Across Color Morphs in the Solitary Ascidian Rhopalaea abdominalis
Source: Int J Microbiol. 2026 Aug 29;2026:1675944. doi: 10.1155/ijm/1675944 (PMC13525377; doi:10.1155/ijm/1675944)
Supplement: Supplementary file 1 [file IJM-2026-1675944-s001.zip › Hutchings_etal_IJM_REV_Supplemental.docx]

**Microbial communities vary by body region but remain consistent across color morphs in the solitary ascidian *Rhopalaea abdominalis***

Brenna Hutchings^1^ ⋅ Susanna López-Legentil^1^ ⋅ Lauren M. Stefaniak^2^ ⋅ Marie L. Nydam^3^ ⋅ Patrick M. Erwin^1^

^1^*Department of Biology & Marine Biology, and Center for Marine Science, University of North Carolina Wilmington, 5600 Marvin K. Moss Lane, Wilmington NC 28409, United States of America*

^2^*Department of Marine Science, Coastal Carolina University, 100 Chanticleer Dr. E., Conway SC 29528, United States of America*

^3^*Life Sciences Concentration, SOKA University of America, 1 University Drive, Aliso Viejo CA 92656, United States of America*

Corresponding author: Patrick M. Erwin

Email: erwinp@uncw.edu

Telephone: 910-619-5401

**Supplementary Materials**

Supplemental Figure Legends

**Figure S1** Stacked bar graphs of relative abundance of microbial domains within each sample for OTU (A) and ASV (B) datasets. Samples are grouped by color morph and body region is indicated by the first letter of the corresponding “Sample ID”: branchial sac (S), gut (G), tunic (T).

**Figure S2** Stacked bar graphs of relative abundance of bacterial phyla within each sample for OTU (A) and ASV (B) datasets. The top ten most abundant phyla within the dataset are shown, with all other phyla represented within the “Other” category. Samples are grouped by color morph and body region is indicated by the first letter of the corresponding “Sample ID”: branchial sac (S), gut (G), tunic (T).

**Figure S3** Stacked bar graphs of relative abundance of bacterial classes within each sample for OTU (A) and ASV (B) datasets. The top ten most abundant classes within the dataset are shown, with all other classes represented within the “Other” category. Samples are grouped by color morph and body region is indicated by the first letter of the corresponding “Sample ID”: branchial sac (S), gut (G), tunic (T).

**Figure S4** Stacked bar graphs of relative abundance of bacterial families within each sample for OTU (A) and ASV (B) datasets. The top ten most abundant families within the dataset are shown, with all other families represented within the “Other” category. Samples are grouped by color morph and body region is indicated by the first letter of the corresponding “Sample ID”: branchial sac (S), gut (G), tunic (T).

**Figure S5** Non-metric multidimensional scaling plots displaying data from OTU (stress=0.102; A) and ASV (stress=0.116; B) datasets. The phylogenetic clade corresponding to each ascidian sample is indicated by color: Clade I (red), Clade II (blue).

**Figure S6** Non-metric multidimensional scaling plots displaying data from OTU (stress= 0.159; A) and ASV (stress=0.161; B) datasets. The collection location corresponding to each ascidian sample is indicated by color: Carrie Bow Cay Forereef (CBC_Fore, red), Carrie Bow Cay Long Reef (CBC_Long, green), Tobacco Cay Deep Reef (Tobacco_Deep, blue), and Tobacco Cay Shallow Reef (Tobacco_Shallow, purple).

Supplemental Tables

**Table S1** Mothur (v.1.43.0) bioinformatics pipeline followed for processing raw sequences.

| Command | Input File Type | Settings |
| --- | --- | --- |
| make.file | .gz |  |
| make.contigs | .fastq | trimoverlap=T, oligos=515f-806r.oligos, pdiffs=4, processors=8 |
| summary.seqs | .fasta |  |
| screen.seqs | .fasta, .groups | maxambig=0, maxlength=300, minlength=200, maxhomop=8 |
| summary.seqs | .fasta |  |
| unique.seqs | .fasta |  |
| align.seqs | .fasta | reference=silva.nr_v132.V4.align, processors=4 |
| summary.seqs | .fasta, .names |  |
| screen.seqs | .fasta, .groups, .names | start=1967, end=11549 |
| summary.seqs | .fasta, .names |  |
| filter.seqs | .fasta | vertical=T, trump=. |
| summary.seqs | .fasta, .names |  |
| pre.cluster | .fasta, .names, .groups | diffs=2 |
| summary.seqs | .fasta, .count_table |  |
| chimera.uchime | .fasta, .count_table | dereplicate=t, reference=self |
| remove.seqs | .fasta, .count_table, .accnos |  |
| summary.seqs | .fasta, .count_table |  |
| classify.seqs | .fasta, .count_table | reference= silva.nr_v132.V4.align, taxonomy=silva.nr_v132.tax, cutoff=60 |
| remove.lineage | .fasta, .count_table, .taxonomy | taxon=Chloroplast-Mitochondria-Eukaryota-unknown |
| summary.seqs | .fasta, .count_table |  |
| filter.seqs | .fasta | vertical=T, trump=. |
| rename.file | .fasta, .count_table, .taxonomy |  |
| dist.seqs | .fasta | cutoff=0.03, processors=4 |
| cluster | .dist, .count_table | cutoff=0.03 |
| remove.rare | .list, .count_table | nseqs=1, label=0.03 |
| classify.otu | .list, .count_table, .taxonomy | label=0.03 |
| get.oturep | .fasta, .count_table, .list | method=abundance |
| make.shared | .list, .count_table |  |
| count.groups | .count_table |  |
| sub.sample | .list, .count_table | persample=t, size=29142 |
| count.groups | .count_table |  |
| list.otulabels | .list |  |
| get.otulabels | .otulabels, .cons.taxonomy |  |
| make.shared | .list, .count_table |  |

**Table S2** QIIME2 (v.2024.2), dada2 (v.1.26.0) bioinformatics pipeline followed for processing raw sequences.

| Command | Input File Type | Settings |
| --- | --- | --- |
| qiime tools import | .manifest.txt | type ‘SampleData[PairedEndSequencesWithQuality]’ |
|  |  | input-format PairedEndFastqManifestPhred33V2 |
| qiime cutadapt trim-paired | .qza | p-front-f GTGCCAGCMGCCGCGGTAA  p-front-r GGACTACHVGGGTWTCTAAT  p-match-read-wildcards  p-match-adapter-wildcards  p-error-rate 0.16  p-discard-untrimmed True |
| qiime demux summarize | .qza |  |
| qiime dada2 denoise-paired | .qza | p-trunc-len-f 240  p-trunc-len-r 180  p-trim-left-f 2  pooling_method independent  chimera_method consensus |
| qiime feature-table merge | .qza |  |
| qiime feature-table merge-seqs | .qza |  |
| qiime feature-classifier classify-sklearn | .qza | i-classifier silva-138-99-515-806-nb-classifier.qza |
| qiime taxa filter-table | .qza | p-exlude Chloroplast,Mitochondria,Eukaryota,Unassigned |
| qiime taxa filter-seqs | .qza | p-exclude Chloroplast,Mitochondria,Eukaryota,Unassigned |
| qiime feature-table rarefy | .qza | p-sampling-depth 29142 |
| qiime metadata tabulate | .qza |  |

**Table S3** Microbial Shannon’s H’ diversity, richness, and Pielou’s evenness for samples processed as OTUs and ASVs. The color morph (purple = P, orange = O, intermediate = I), phylogentic clade (Clade I = I, Clade II = II, n.a. = not available) and body region of origin (tunic = T, branchial sac = S, gut = G) of each sample is indicated. Seawater samples have no entry listed in color and region columns.

| Sample ID | | Color | Clade | Region | OTU | | | ASV | | |
| --- | --- | --- | --- | --- | --- | --- | --- | --- | --- | --- |
|  |  | | | | Diversity | Richness | Evenness | Diversity | Richness | Evenness |
| 7Jul23 1-2A | | P | I | T | 4.452 | 497 | 0.717 | 4.808 | 514 | 0.770 |
|  | |  |  | S | 2.622 | 293 | 0.462 | 3.383 | 329 | 0.584 |
| 7Jul23 1-2B | | P | n.a. | S | 2.878 | 385 | 0.483 | 3.591 | 393 | 0.601 |
|  | |  |  | G | 2.082 | 108 | 0.445 | 2.493 | 89 | 0.555 |
| 7Jul23 1-2C | | P | I | S | 2.832 | 200 | 0.534 | 3.219 | 190 | 0.614 |
|  | |  |  | G | 2.323 | 172 | 0.451 | 2.856 | 148 | 0.572 |
| 7Jul23 1-3A | | O | II | S | 2.205 | 204 | 0.415 | 2.603 | 259 | 0.469 |
| 7Jul23 1-3B | | O | II | T | 0.733 | 170 | 0.143 | 0.626 | 166 | 0.122 |
|  | |  |  | S | 2.728 | 618 | 0.425 | 3.559 | 650 | 0.550 |
|  | |  |  | G | 1.735 | 171 | 0.337 | 2.731 | 181 | 0.525 |
| 7Jul23 2-7 | | O | II | T | 4.980 | 1511 | 0.680 | 5.294 | 1039 | 0.762 |
|  | |  |  | S | 2.906 | 297 | 0.510 | 3.709 | 230 | 0.682 |
| 7Jul23 2-AW | |  |  |  | 4.086 | 886 | 0.602 | 4.953 | 923 | 0.725 |
| 7Jul23 2-BW | |  |  |  | 3.935 | 847 | 0.584 | 4.792 | 904 | 0.704 |
| 7Jul23 2-CW | |  |  |  | 3.965 | 876 | 0.585 | 4.953 | 979 | 0.719 |
| 8Jul23 1-3A | | O | I | T | 4.041 | 498 | 0.651 | 4.729 | 288 | 0.835 |
|  | |  |  | S | 2.830 | 435 | 0.466 | 3.099 | 468 | 0.504 |
|  | |  |  | G | 5.776 | 3712 | 0.703 | 5.790 | 1882 | 0.768 |
| 8Jul23 1-3B | | O | II | T | 5.541 | 2902 | 0.695 | 5.772 | 1830 | 0.768 |
|  | |  |  | S | 1.778 | 185 | 0.340 | 1.712 | 183 | 0.329 |
|  | |  |  | G | 5.367 | 2046 | 0.704 | 5.834 | 2675 | 0.739 |
| 8Jul23 1-6A | | I | I | S | 2.945 | 436 | 0.485 | 3.273 | 452 | 0.535 |
|  | |  |  | G | 1.624 | 191 | 0.309 | 2.746 | 156 | 0.544 |
| 8Jul23 1-6B | | I | II | T | 5.632 | 3451 | 0.691 | 5.684 | 1808 | 0.758 |
|  | |  |  | S | 2.695 | 904 | 0.400 | 2.967 | 929 | 0.434 |
|  | |  |  | G | 0.933 | 63 | 0.225 | 0.979 | 67 | 0.233 |
| 8Jul23 1-AW | |  |  |  | 4.003 | 812 | 0.597 | 4.940 | 989 | 0.716 |
| 8Jul23 1-BW | |  |  |  | 3.839 | 770 | 0.578 | 4.843 | 940 | 0.707 |
| 8Jul23 1-CW | |  |  |  | 3.849 | 804 | 0.575 | 4.825 | 917 | 0.707 |
| 8Jul23 2-2A | | I | I | T | 1.000 | 47 | 0.260 | 0.883 | 34 | 0.250 |
|  | |  |  | S | 2.191 | 230 | 0.403 | 2.706 | 213 | 0.505 |
|  | |  |  | G | 2.050 | 94 | 0.451 | 2.179 | 99 | 0.474 |
| 8Jul23 2-2B | | I | I | T | 4.199 | 170 | 0.818 | 4.350 | 113 | 0.920 |
|  | |  |  | S | 2.293 | 382 | 0.386 | 2.309 | 415 | 0.383 |
|  | |  |  | G | 4.036 | 172 | 0.784 | 4.344 | 111 | 0.922 |
| 8Jul23 2-3 | | P | I | T | 4.257 | 245 | 0.774 | 4.352 | 149 | 0.870 |
|  | |  |  | S | 1.506 | 255 | 0.272 | 1.531 | 279 | 0.272 |
|  | |  |  | G | 2.674 | 419 | 0.443 | 3.257 | 383 | 0.548 |

**Table S4** Mean, t stat, t critical, and p-value for paired t-test comparing diversity, richness, and evenness between OTU and ASV datasets. Significant values in bold.

| Comparison | Mean (OTU) | Mean (ASV) | t stat | t critical | p-value |
| --- | --- | --- | --- | --- | --- |
| Diversity | 3.145 | 3.600 | 7.569 | 1.687 | **2.510x10^-9^** |
| Richness | 696.263 | 588.789 | 1.467 | 1.687 | 0.075 |
| Evenness | 0.510 | 0.597 | 8.799 | 1.687 | **6.640x10^-11^** |

**Table S5** Mean, t stat, t critical (t crit), and p-value (p) for t-tests comparing diversity, richness, and evenness across COI clades for OTU and ASV datasets.

|  | **OTU** | | | | |  | **ASV** | | | | |
| --- | --- | --- | --- | --- | --- | --- | --- | --- | --- | --- | --- |
| Comparison | Mean (Clade I) | Mean (Clade II) | t stat | t crit | p |  | Mean (Clade I) | Mean (Clade II) | t stat | t crit | p |
| Diversity | 2.981 | 3.103 | -0.220 | 2.048 | 0.827 |  | 3.323 | 3.456 | -0.236 | 2.048 | 0.815 |
| Richness | 469.333 | 1043.500 | -1.580 | 2.048 | 0.125 |  | 345.722 | 834.750 | -1.850 | 2.145 | 0.086 |
| Evenness | 0.521 | 0.464 | 0.826 | 2.048 | 0.416 |  | 0.604 | 0.531 | 0.926 | 2.048 | 0.362 |

**Table S6** R^2^, F-statistic (F) and p-value (p) from PERMANOVAs comparing microbial composition by host phylogenetic clade (I, II) for OTU and ASV datasets.

|  | OTU | | | | ASV | | | |
| --- | --- | --- | --- | --- | --- | --- | --- | --- |
| Clade | R^2^ | F | p | p-adj | R^2^ | F | p | p-adj |
| I x II | 0.024 | 0.691 | 0.846 | 0.846 | 0.033 | 0.965 | 0.511 | 0.511 |

**Table S7** R^2^, F-statistic (F), and FDR-adjusted p-values (p-adj) from pairwise PERMANOVA comparing microbial composition by collection location for OTU and ASV datasets. CBC_Fore = Carrie Bow Cay Forereef, CBC_Long = Carrie Bow Cay Long Reef, Tobacco_Deep = Tobacco Cay Deep Reef, and Tobacco_Shallow = Tobacco Cay Shallow Reef.

|  | OTU | | | ASV | | |
| --- | --- | --- | --- | --- | --- | --- |
| Pairwise Location Comparison | R^2^ | F | p-adj | R^2^ | F | p-adj |
| CBC_Fore vs CBC_Long | 0.097 | 1.072 | 0.335 | 0.085 | 0.926 | 0.420 |
| CBC_Fore vs Tobacco_Deep | 0.089 | 1.860 | 0.087 | 0.091 | 1.902 | 0.108 |
| CBC_Fore vs Tobacco_Shallow | 0.096 | 1.807 | 0.087 | 0.104 | 1.965 | 0.108 |
| CBC_Long vs Tobacco_Deep | 0.094 | 1.141 | 0.335 | 0.083 | 0.998 | 0.420 |
| CBC_Long vs Tobacco_Shallow | 0.115 | 1.174 | 0.167 | 0.108 | 1.094 | 0.386 |
| Tobacco_Deep vs Tobacco_Shallow | 0.075 | 1.460 | 0.160 | 0.071 | 1.368 | 0.254 |

**Table S8** Number of core OTUs and ASVs by body region (branchial sac (S), gut (G), tunic (T)) within each color morph (CM; purple (P), orange (O), intermediate (I)) and combination of color morphs. The number of core OTUs and ASVs by color morph only is also indicated (Only CM).

|  | OTU | | | | ASV | | | |
| --- | --- | --- | --- | --- | --- | --- | --- | --- |
| CM | S | G | T | Only CM | S | G | T | Only CM |
| O | 30 | 40 | 22 | 5 | 21 | 21 | 7 | 3 |
| P | 27 | 44 | 34 | 1 | 19 | 38 | 20 | 0 |
| I | 39 | 1 | 1 | 0 | 32 | 0 | 1 | 0 |
| O x P | 14 | 15 | 6 | 1 | 8 | 12 | 2 | 0 |
| O x I | 16 | 1 | 1 | 0 | 11 | 0 | 0 | 0 |
| P x I | 17 | 1 | 0 | 0 | 10 | 0 | 0 | 0 |
| O x P x I | 12 | 1 | 0 | 0 | 8 | 0 | 0 | 0 |

**Table S9** The top five most abundant core OTU identification numbers (#) and lowest known taxonomic rank (ID; d: domain, p: phylum, c: class, o: order, f: family, g: genus) by body region for each color morph (CM: purple (P), orange (O), intermediate (I)) and combination of color morphs. Cells with dashed lines indicate less than five core OTUs were identified for a given category.

|  | Core OTUs | | | | | |
| --- | --- | --- | --- | --- | --- | --- |
|  | Branchial Sac | | Gut | | Tunic | |
| CM | # | ID | # | ID | # | ID |
| O | 1 | f. Vibrionaceae | 3 | g. *Synechococcus_CC9902* | 1 | f. Vibrionaceae |
|  | 2 | g. *Endozoicomonas* | 1 | f. Vibrionaceae | 21 | g. *Pseudoalteromonas* |
|  | 6 | g. *Alteromonas* | 20 | g. *Roseimarinus* | 146 | f. Shewanellaceae |
|  | 22 | g. *Photobacterium* | 26 | g. *Synechococcus_CC9902* | 22 | g. *Photobacterium* |
|  | 17 | g. *Ferrimonas* | 2 | g. *Endozoicomonas* | 117 | g. *Thalassomonas* |
| P | 1 | f. Vibrionaceae | 3 | g. *Synechococcus_CC9902* | 47 | c. Bacteroidia |
|  | 8 | g. *Arcobacter* | 9 | g. *Roseimarinus* | 14 | g. *Propionigenium* |
|  | 2 | g. *Endozoicomonas* | 18 | f. Vibrionaceae | 1 | f. Vibrionaceae |
|  | 17 | g. *Ferrimonas* | 14 | g. *Propionigenium* | 130 | f. SM23-30 (Phycisphaerae) |
|  | 21 | g. *Pseudoalteromonas* | 20 | g. *Roseimarinus* | 107 | o. Bacteroidales |
| I | 6 | g. *Alteromonas* | 1 | f. Vibrionaceae | 70 | g. *Aestuariibacter* |
|  | 2 | g. *Endozoicomonas* | - | - | - | - |
|  | 4 | f. Thioglobaceae | - | - | - | - |
|  | 1 | f. Vibrionaceae | - | - | - | - |
|  | 19 | g. *Endozoicomonas* | - | - | - | - |
| O x P | 1 | f. Vibrionaceae | 3 | g. *Synechococcus_CC9902* | 1 | f. Vibrionaceae |
|  | 2 | g. *Endozoicomonas* | 1 | f. Vibrionaceae | 14 | g. *Propionigenium* |
|  | 6 | g. *Alteromonas* | 18 | f. Vibrionaceae | 67 | g. *Ruegeria* |
|  | 17 | g. *Ferrimonas* | 20 | g. *Roseimarinus* | 82 | g. *Candidatus Nitrosopumilus* |
|  | 21 | g. *Pseudoalteromonas* | 14 | g. *Propionigenium* | 217 | f. Rhodobacteraceae |
| O x I | 2 | g. *Endozoicomonas* | 1 | f. Vibrionaceae | 70 | g. *Aestuariibacter* |
|  | 1 | f. Vibrionaceae | - | - | - | - |
|  | 6 | g. *Alteromonas* | - | - | - | - |
|  | 17 | g. *Ferrimonas* | - | - | - | - |
|  | 22 | g. *Photobacterium* | - | - | - | - |
| P x I | 1 | f. Vibrionaceae | 1 | f. Vibrionaceae | - | - |
|  | 2 | g. *Endozoicomonas* | - | - | - | - |
|  | 6 | g. *Alteromonas* | - | - | - | - |
|  | 8 | g. *Arcobacter* | - | - | - | - |
|  | 19 | g. *Endozoicomonas* | - | - | - | - |
| O x P x I | 1 | f. Vibrionaceae | 1 | f. Vibrionaceae | - | - |
|  | 2 | g. *Endozoicomonas* | - | - | - | - |
|  | 6 | g. *Alteromonas* | - | - | - | - |
|  | 17 | g. *Ferrimonas* | - | - | - | - |
|  | 21 | g. *Pseudoalteromonas* | - | - | - | - |

**Table S10** The top five most abundant core ASV identification numbers (#) and lowest known taxonomic rank (ID; d: domain, p: phylum, c: class, o: order, f: family, g: genus) by body region for each color morph (CM: purple (P), orange (O), intermediate (I)) and combination of color morphs. Cells with dashed lines indicate less than five core ASVs were identified for a given category.

|  | Core ASVs | | | | | |
| --- | --- | --- | --- | --- | --- | --- |
|  | Branchial Sac | | Gut | | Tunic | |
| CM | # | ID | # | ID | # | ID |
| O | 2 | g. *Endozoicomonas* | 6 | g. *Vibrio* | 6 | g. *Vibrio* |
|  | 3 | g. *Alteromonas* | 10 | g. *Synechococcus_CC9902* | 21 | g. *Vibrio* |
|  | 6 | g. *Vibrio* | 14 | g. *Synechococcus_CC9902* | 24 | g. *Photobacterium* |
|  | 8 | g. *Vibrio* | 16 | g. *Synechococcus_CC9902* | 176 | g. *Vibrio* |
|  | 21 | g. *Vibrio* | 25 | g. *Synechococcus_CC9902* | 68 | f. Rhodobacteraceae |
| P | 9 | f. Arcobacteraceae | 7 | g. *Roseimarinus* | 28 | c. Bacteroidia |
|  | 8 | g. *Vibrio* | 18 | f. Vibrionaceae | 6 | g. *Vibrio* |
|  | 2 | g. *Endozoicomonas* | 15 | g. *Propionigenium* | 88 | f. SM23-30 (Phycisphaerae) |
|  | 23 | g. *Pseudoalteromonas* | 10 | g. *Synechococcus_CC9902* | 42 | g. *Propionigenium* |
|  | 31 | g. *Ferrimonas* | 14 | g. *Synechococcus_CC9902* | 68 | f. Rhodobacteraceae |
| I | 3 | g. *Alteromonas* | - |  | 150 | g. *Aestuariibacter* |
|  | 2 | g. *Endozoicomonas* | - |  | - |  |
|  | 8 | g. *Vibrio* | - |  | - |  |
|  | 6 | g. *Vibrio* | - |  | - |  |
|  | 78 | o. Rhizobiales | - |  | - |  |
| O x P | 2 | g. *Endozoicomonas* | 10 | g. *Synechococcus_CC9902* | 6 | g. *Vibrio* |
|  | 3 | g. *Alteromonas* | 15 | g. *Propionigenium* | 68 | f. Rhodobacteraceae |
|  | 8 | g. *Vibrio* | 18 | f. Vibrionaceae | - |  |
|  | 23 | g. *Pseudoalteromonas* | 6 | g. *Vibrio* | - |  |
|  | 31 | g. *Ferrimonas* | 14 | g. *Synechococcus_CC9902* | - |  |
| O x I | 2 | g. *Endozoicomonas* | - |  | - |  |
|  | 3 | g. *Alteromonas* | - |  | - |  |
|  | 8 | g. *Vibrio* | - |  | - |  |
|  | 6 | g. *Vibrio* | - |  | - |  |
|  | 24 | g. *Photobacterium* | - |  | - |  |
| P x I | 3 | g. *Alteromonas* | - |  | - |  |
|  | 8 | g. *Vibrio* | - |  | - |  |
|  | 2 | g. *Endozoicomonas* | - |  | - |  |
|  | 23 | g. *Pseudoalteromonas* | - |  | - |  |
|  | 31 | g. *Ferrimonas* | - |  | - |  |
| O x P x I | 2 | g. *Endozoicomonas* | - |  | - |  |
|  | 3 | g. *Alteromonas* | - |  | - |  |
|  | 8 | g. *Vibrio* | - |  | - |  |
|  | 23 | g. *Pseudoalteromonas* | - |  | - |  |
|  | 31 | g. *Ferrimonas* | - |  | - |  |

**Table S11** OTU and ASV identification numbers (#), p-values (p), FDR-adjusted p-values (p-adj), and lowest known taxonomic rank (ID) for the top five most abundant pairwise comparisons made by body region (branchial sac (S), gut (G), tunic (T), seawater (SW)) and color morph (purple (P), orange (O), intermediate (I), seawater (SW)).

|  | OTU | | | | ASV | | | |
| --- | --- | --- | --- | --- | --- | --- | --- | --- |
| Pair | # | p | p-adj | ID | # | p | p-adj | ID |
| S x G | 2 | 4.74x10^-6^ | 6.18x10^-4^ | g. *Endozoicomonas* | 2 | 1.11x10^-5^ | 8.18x10^-4^ | g. *Endozoicomonas* |
|  | 6 | 8.51x10^-8^ | 1.51x10^-5^ | g. *Alteromonas* | 3 | 2.48x10^-8^ | 2.60x10^-6^ | g. *Alteromonas* |
|  | 10 | 6.41x10^-5^ | 6.27x10^-3^ | g. *Endozoicomonas* | 8 | 4.86x10^-15^ | 1.18x10^-12^ | g. *Vibrio* |
|  | 19 | 1.59x10^-7^ | 2.69x10^-5^ | g. *Endozoicomonas* | 10 | 5.13x10^-4^ | 0.029 | g. *Synechococcus CC9902* |
|  | 21 | 1.24x10^-4^ | 1.10x10^-2^ | g. *Pseudoalteromonas* | 14 | 5.75x10^-4^ | 0.032 | g. *Synechococcus CC9902* |
| S x T | 2 | 5.19x10^-12^ | 2.86x10^-9^ | g. *Endozoicomonas* | 2 | 1.07x10^-11^ | 1.99x10^-9^ | g. *Endozoicomonas* |
|  | 5 | 8.88x10^-5^ | 9.58x10^-3^ | f. S25-593 (Rickettsiales) | 3 | 3.35x10^-9^ | 4.77x10^-7^ | g. *Alteromonas* |
|  | 6 | 2.03x10^-8^ | 5.26x10^-6^ | g. *Alteromonas* | 4 | 1.09x10^-4^ | 7.56x10^-3^ | f. S25-593 (Rickettsiales) |
|  | 8 | 1.96x10^-4^ | 1.97x10^-2^ | g. *Arcobacter* | 7 | 5.96x10^-4^ | 0.035 | g. *Roseimarinus* |
|  | 9 | 1.02x10^-4^ | 1.09x10^-2^ | g. *Roseimarinus* | 8 | 1.27x10^-16^ | 4.81x10^-14^ | g. *Vibrio* |
| G x T | 2 | 3.43x10^-5^ | 4.35x10^-3^ | g. *Endozoicomonas* | 2 | 3.53x10^-5^ | 0.003 | g. *Endozoicomonas* |
|  | 5 | 3.74x10^-5^ | 4.69x10^-3^ | f. S25-593 (Rickettsiales) | 4 | 3.76x10^-5^ | 0.003 | f. S25-593 (Rickettsiales) |
|  | 9 | 7.23x10^-7^ | 1.33x10^-4^ | g. *Roseimarinus* | 7 | 8.93x10^-7^ | 8.90x10^-5^ | g. *Roseimarinus* |
|  | 15 | 2.56x10^-4^ | 2.50x10^-2^ | g. *Candidatus* Hepatoplasma | 20 | 2.74x10^-4^ | 0.018 | g. *Candidatus* Hepatoplasma |
|  | 18 | 5.42x10^-4^ | 4.60x10^-2^ | f. Vibrionaceae | 25 | 2.99x10^-4^ | 0.019 | g. *Synechococcus CC9902* |
| S x SW | 1 | 1.87x10^-7^ | 1.18x10^-5^ | f. Vibrionaceae | 2 | 3.77x10^-10^ | 1.87x10^-8^ | g. *Endozoicomonas* |
|  | 2 | 3.85x10^-10^ | 4.66x10^-8^ | g. *Endozoicomonas* | 4 | 5.03x10^-4^ | 0.010 | f. S25-593 (Rickettsiales) |
|  | 5 | 4.24x10^-4^ | 1.25x10^-2^ | f. S25-593 (Rickettsiales) | 6 | 5.38x10^-4^ | 0.011 | g. *Vibrio* |
|  | 7 | 8.46x10^-6^ | 3.87x10^-4^ | f. Clade I (SAR11) | 7 | 2.12x10^-3^ | 0.040 | g. *Roseimarinus* |
|  | 8 | 2.65x10^-4^ | 8.34x10^-3^ | g. *Arcobacter* | 8 | 5.54x10^-15^ | 4.80x10^-13^ | g. *Vibrio* |
| G x SW | 2 | 4.66x10^-4^ | 1.37x10^-2^ | g. *Endozoicomonas* | 2 | 2.35x10^-4^ | 0.005 | g. *Endozoicomonas* |
|  | 5 | 1.74x10^-4^ | 5.78x10^-3^ | f. S25-593 (Rickettsiales) | 3 | 8.50x10^-4^ | 0.017 | g. *Alteromonas* |
|  | 6 | 5.87x10^-4^ | 1.70x10^-2^ | g. *Alteromonas* | 4 | 1.74x10^-4^ | 0.004 | f. S25-593 (Rickettsiales) |
|  | 7 | 1.64x10^-7^ | 1.06x10^-5^ | f. Clade I (SAR11) | 7 | 6.45x10^-6^ | 1.74x10^-4^ | g. *Roseimarinus* |
|  | 9 | 4.41x10^-6^ | 2.12x10^-4^ | g. *Roseimarinus* | 11 | 1.86x10^-6^ | 5.46x10^-5^ | g. *Prochlorococcus* MIT9313 |
| T x SW | 1 | 5.46x10^-4^ | 4.62x10^-2^ | f. Vibrionaceae | 3 | 1.22x10^-4^ | 0.003 | g. *Alteromonas* |
|  | 6 | 1.42x10^-4^ | 1.46x10^-2^ | g. *Alteromonas* | 11 | 4.07x10^-6^ | 1.13x10^-4^ | g. *Prochlorococcus* MIT9313 |
|  | 7 | 2.37x10^-6^ | 3.91x10^-4^ | f. Clade I (SAR11) | 13 | 1.29x10^-5^ | 3.35x10^-4^ | f. Clade II (SAR11) |
|  | 11 | 1.24x10^-5^ | 1.67x10^-3^ | f. Clade II (SAR11) | 16 | 4.80x10^-5^ | 0.001 | g. *Synechococcus CC9902* |
|  | 13 | 4.33x10^-6^ | 6.49x10^-4^ | g. *Prochlorococcus* MIT9313 | 19 | 4.47x10^-7^ | 1.45x10^-5^ | f. Clade I (SAR11) |
| I x O | - | - | - | - | 36 | 5.90x10^-4^ | 0.036 | g. *Vibrio* |
|  | - | - | - | - | 47 | 3.84x10^-4^ | 0.024 | g. *Vibrio* |
|  | - | - | - | - | 176 | 1.56x10^-4^ | 0.012 | g. *Vibrio* |
|  | - | - | - | - | 593 | 4.32x10^-4^ | 0.027 | g. *Psychrosphaera* |
| I x P | 395 | 3.89x10^-4^ | 3.58x10^-2^ | g. *Propionigenium* | 15 | 7.02x10^-4^ | 0.043 | g. *Propionigenium* |
|  | - | - | - | - | 172 | 5.76x10^-4^ | 0.036 | g. *Propionigenium* |
| O x P | - | - | - | - | 593 | 7.78x10^-4^ | 0.047 | g. *Psychrosphaera* |
| I x SW | 7 | 1.04x10^-6^ | 5.72x10^-5^ | f. Clade I (SAR11) | 11 | 1.19x10^-6^ | 3.58x10^-5^ | g. *Prochlorococcus* MIT9313 |
|  | 11 | 4.84x10^-6^ | 2.31x10^-4^ | f. Clade II (SAR11) | 13 | 2.56x10^-6^ | 7.26x10^-5^ | f. Clade II (SAR11) |
|  | 13 | 1.53x10^-6^ | 8.22x10^-5^ | g. *Prochlorococcus* MIT9313 | 16 | 0.001 | 0.030 | g. *Synechococcus CC9902* |
|  | 29 | 7.94x10^-8^ | 5.57x10^-6^ | o. SAR86 Clade | 19 | 5.52x10^-8^ | 1.99x10^-6^ | f. Clade I (SAR11) |
|  | 31 | 7.21x10^-8^ | 5.11x10^-6^ | f. AEGEAN-169 Marine Group | 26 | 4.86x10^-7^ | 1.56x10^-5^ | f. Clade I (SAR11) |
| O x SW | 1 | 6.31x10^-6^ | 2.96x10^-4^ | f. Vibrionaceae | 6 | 3.32x10^-5^ | 8.35x10^-4^ | g. *Vibrio* |
|  | 7 | 9.17x10^-6^ | 4.21x10^-4^ | f. Clade I (SAR11) | 11 | 7.05x10^-6^ | 1.88x10^-4^ | g. *Prochlorococcus* MIT9313 |
|  | 11 | 7.49x10^-6^ | 3.46x10^-4^ | f. Clade II (SAR11) | 13 | 3.73x10^-6^ | 1.03x10^-4^ | f. Clade II (SAR11) |
|  | 13 | 6.13x10^-6^ | 2.88x10^-4^ | g. *Prochlorococcus* MIT9313 | 16 | 5.38x10^-4^ | 0.011 | g. *Synechococcus CC9902* |
|  | 14 | 7.17x10^-4^ | 2.11x10^-2^ | g. *Propionigenium* | 19 | 5.90x10^-8^ | 2.11x10^-6^ | f. Clade I (SAR11) |
| P x SW | 1 | 1.28x10^-4^ | 4.56x10^-3^ | f. Vibrionaceae | 6 | 0.002 | 0.040 | g. *Vibrio* |
|  | 7 | 2.59x10^-6^ | 1.30x10^-4^ | f. Clade I (SAR11) | 11 | 2.40x10^-5^ | 6.07x10^-4^ | g. *Prochlorococcus* MIT9313 |
|  | 11 | 2.00x10^-6^ | 1.04x10^-4^ | f. Clade II (SAR11) | 13 | 1.08x10^-6^ | 3.29x10^-5^ | f. Clade II (SAR11) |
|  | 13 | 3.73x10^-5^ | 1.52x10^-3^ | g. *Prochlorococcus* MIT9313 | 15 | 5.39x10^-5^ | 0.001 | g. *Propionigenium* |
|  | 14 | 1.03x10^-4^ | 3.73x10^-3^ | g. *Propionigenium* | 18 | 0.001 | 0.026 | f. Vibrionaceae |

**Figure S1**

**Figure S2**

**Figure S3**

**Figure S4**

**Figure S5**

**Figure S6**
